# Stem cell-based embryo models as a tool for reproductive biology

**DOI:** 10.1093/molehr/gaag030

**Published:** 2026-05-12

**Authors:** Alfonso Martinez Arias, Andre Dias, Maneesha Inamdar

**Affiliations:** MELIS, Universitat Pompeu Fabra, Barcelona, Spain; MELIS, Universitat Pompeu Fabra, Barcelona, Spain; Centre for Research Application and Training in Embryology (CReATE), Institute for Stem Cell Science and Regenerative Medicine (BRIC-InStem), Bangalore, India; Jawaharlal Nehru Centre for Advanced Scientific Research (JNCASR), Bangalore, India

**Keywords:** embryology, stem cells, implantation, gene expression, pregnancy

## Abstract

ART has transformed clinical practice but still faces modest success rates, poorly defined implantation conditions, and persistent gaps in understanding early human development. At the same time, research in human reproductive biology remains strikingly underfunded relative to its medical and social impact. Over the last decade, advances in human pluripotent stem cell (PSC) biology have generated a suite of technologies that can recapitulate early stages of human development from blastocyst formation to early gastrulation *in vitro*. Here, we review how such models can be harnessed to interrogate lineage specification, embryo–endometrium crosstalk, the origins of aneuploidy and implantation failure, and the developmental basis of placental and yolk sac disorders, with a focus on primates. We argue that integrating stem cell–derived embryo models into reproductive research agendas offers a tractable experimental framework to improve ART outcomes and address major unmet needs in human reproductive health.

## Introduction

It is almost 50 years since Louise Brown was born in Oldham (UK) on 25 July 1978: the first baby not to be conceived in a womb and a historical landmark forever associated with IVF. A few months later, a second baby, Alistair McDonald, was born from the same procedure, proving that there was no miracle; the technique worked. Since that date, IVF has become a wide range of techniques under the acronym ART that have brought over 18 million babies into this world, fulfilling the hopes of many women and couples. The feat was the result of years of work by the joint efforts of Robert Edwards, Jean Purdy, and Patrick Steptoe (Edwards and Steptoe, 2012; Gosden, 2021).

While IVF is rightly celebrated for what it has achieved in the clinic, its relationship with basic research, which played a major role in its development, is less appreciated. A reason for this probably lies in the perception of ART as a suite of technologies associated with fertility clinics rather than with research laboratories. The difficulty in bridging the two seems to be part of the history of the field. Edwards failed twice to secure funding for joint ventures between the two fields in the UK and Bourne Hall in Cambridgeshire, the first IVF clinic in the world, was built with private funding (Johnson *et al.*, 2010; Gosden, 2021).

ART has brought out into the open a number of issues associated with human reproduction that historically were not of much biomedical interest: female and male reproductive health, fertility and contraception, pregnancy loss, abortion, and teratology. These issues now have a significant social impact and demand attention. Clear examples are the need to improve ART success rates that remain low, in the 40% range and going down to 30% after 40 years of maternal age (Wade *et al.*, 2015; HFEA, 2025), or the identification of good morulae or blastocysts to implant which remains challenging, and even making use of the information gathered over decades, success rates are about 60%, or 70% when AI is used (Salih *et al.*, 2023). Furthermore, conditions for successful blastocyst implantation, a moment at which most conceptions fail, remain ill-defined. In contrast with the importance and urgency of these needs, research on human reproduction is underfunded in comparison to other areas of biomedical research. For instance, only about 7–10% (2% specific reproductive health minus cancers) of the NIH budget is dedicated to human reproduction (National Academies of Sciences, Engineering and Medicine, 2024), and this before the recent US budget cuts in research (Xu, 2025). It is also surprising that while there is a National Institute of Ageing in the USA and much private funding in this area, nothing of the kind exists in the area of reproductive biology. The situation is not better in Europe, where the EU allocates about 1% of its research budget to sexual and reproductive health (Countdown 2030 Europe, 2021; Lenz *et al.*, 2025).

This underfunding is surprising, for if one looks at the early history of IVF, one can see that its success is tightly associated with basic research and would not have happened without a deep dive into fundamental reproductive and developmental biology (Johnson, 2019). Research into oocyte maturation, sperm capacitation, *in vitro* blastocyst development, and maturation were all required for the success of the technique. Moreover, human reproduction has become a focal point of contemporary political and societal debate in many countries, with topics such as abortion and assisted reproduction requiring proper and advanced scientific grounding. Strengthening basic research is, therefore, not only scientifically justified but socially necessary.

An area where basic research is already opening transformative possibilities is the rapidly evolving field of pluripotent stem cells (PSCs). Over the last 10 years, developments in this area have triggered new perspectives in the study of human embryos, in particular the early stages of human development. This has been fuelled in particular by the discovery of the ability of PSCs to self-organize into structures that mimic blastocysts and, in some cases, also reflect gastrulation, the process that lays down the body plan (Wu and Wang, 2026). The foundation of this work has been made through research on mouse embryos, but increasingly, systems are being developed with human PSCs that can address specific questions of human biology.

Here, we review the current state of the field from the perspective of what it can do for reproductive health. We will focus on embryonic development and restrict ourselves to primates, human and non-human, and refer to mouse only as necessary.

## Human development: from zygote to body plan

It is underappreciated that research on the early stages of human development has its roots in the development of IVF. The work of Edwards in the 1960s built on a small tradition of interest in human development in the USA, underpinned by work at the Carnegie Institute in Baltimore (Noe, 2004; de Bakker *et al.*, 2016). Despite some early hints of successful IVF, it was the sustained efforts of Edwards and his colleagues, Jean Purdy and Patrick Steptoe, that delivered a successful method (Johnson, 2019). A close look at the history of IVF reveals three phases, each invested in basic research.

**Figure 1.**
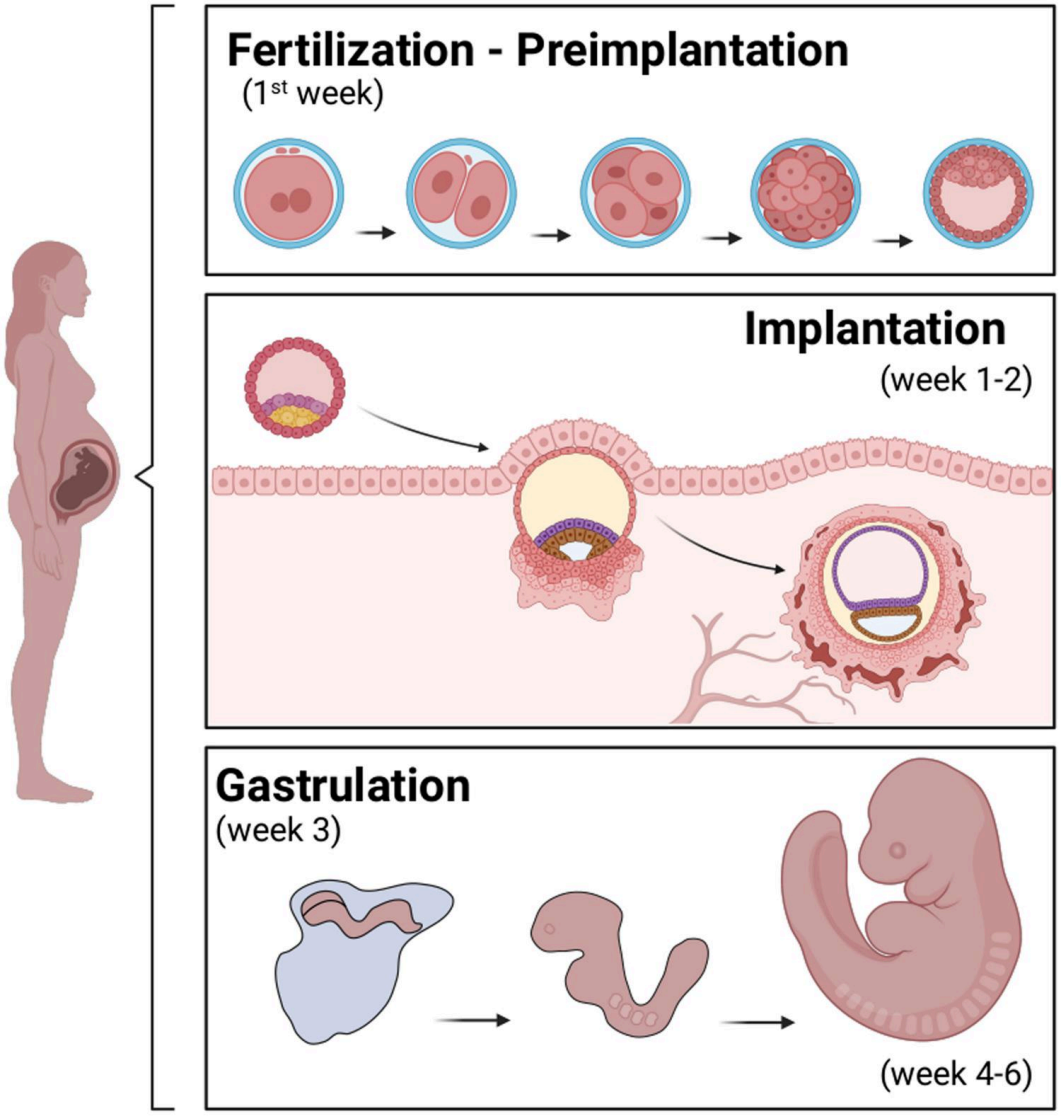
**Stages of human development during the first 6 weeks post-fertilization**. For details, see text. The top panel shows the formation of the blastocyst through the cleavage of the zygote. The middle panel shows the implantation of the blastocyst in the uterus with the expansion and differentiation of the trophectoderm and the remodelling of the endometrium. The bottom panel focuses on the embryo (brown) and shows its development from week 3 through to weeks 4 and 6. Created in BioRender. Dias, A. (2026). https://BioRender.com/js6cxnt.

The first one concerned understanding the maturation of the eggs and the sperm and finding a way to bring them together in a dish in a productive manner. This took a long time and was built on top of work that Edwards and others had done in mice and in rabbits (Johnson, 2019). Although there was a report of a successful fertilization in humans with one or two divisions, it was the work of Bavister and Edwards (Edwards *et al.*, 1969) that pushed the work forward. The second phase required the development of the fertilized egg in the laboratory until the blastocyst stage (Edwards *et al.*, 1969; Steptoe *et al.*, 1971). Taking advantage of the free-floating growth of the zygote as it divides rolling down the fallopian tubes, this work opened up the door to our current understanding of the early stages of human development and, indirectly, to the development of PSCs, which would not have been possible without the ability to culture blastocysts (Thomson *et al.*, 1998). Finally, the last phase was the implantation of the morula and the blastocyst. Here, at the time, there was little that Edwards and his team could do, and it was left to nature, but, as described below, this is now an important focus of research. The techniques associated with each of these phases have enabled a great deal of biology and led to a clear picture of these early stages of human development (Fig. 1).

After the sperm fertilizes the egg to produce the zygote, the fusion of the two pronuclei leads to the first cleavages. Over the next 2 days, as the number of cells increases, the nuclei are epigenetically reprogrammed, chromatin remodelling resets the genome, the zygotic gene activation (ZGA) starts, and successive mitosis generate a group of 8–12 cells. At this time, the cells compact and sort into a structure with an outer epithelial layer, the trophectoderm (TE), that will give rise to the placenta, and an inner core of non-polarized cells, the inner cell mass (ICM). Proliferation continues at a slow pace, and during the fourth day, trophectoderm cells pump liquid into the ICM to generate an inner cavity that pushes the ICM to one side. As this is going on, the ICM differentiates into an inner core, the embryonic cells tucked against the TE, and an outer coat facing the cavity, the primitive endoderm (PrEnd) that will form the yolk sac.

While the sequence of events that generate the blastocyst is similar in mouse and humans, it has become clear that human embryos are more sensitive than mice to mechanical damage, and this leads to a high frequency of aneuploids and embryo loss (Mantzouratou and Delhanty, 2011; Firmin *et al.*, 2024). It has been suggested that this is associated with the activity of adhesion and the cytoskeleton during this process (Pelzer *et al.*, 2023; Firmin *et al.*, 2024).

Once the blastocyst hatches, it attaches to the uterine wall and initiates implantation. This is a process where mouse and human differ dramatically (Yoshinaga, 2018; Dimova *et al.*, 2025). In both cases, the process is guided by TE cells, but whereas in the case of humans, the process is led by TE cells associated with the embryonic cells (polar TE), in the mouse, it is led by the opposite, mural TE cells. In addition, whereas the mouse has a superficial implantation with little uterine invasion, the implantation of the human blastocyst is very deep, the deepest known amongst primates (Ross and Boroviak, 2020; Siriwardena and Boroviak, 2022).

In humans, implantation lasts 1 week and is associated with dramatic changes in cell composition and organization of the three lineages (Yoshinaga, 2018; Bondarenko and Turco, 2025; Dimova *et al.*, 2025). The TE differentiates into cytotrophoblasts and syncytiotrophoblasts, which lead the interaction with the endometrium with a large increase in numbers and the formation of a syncytium that promotes a reorganization of the uterine vasculature to nurture the embryo (Aplin and Ruane, 2017; Ruane *et al.*, 2022). Later, the interactions between trophoblasts and endometrium will form the placenta (Hamilton and Boyd, 1960). The cytotrophoblasts have an important role in providing progenitors for the syncytiotrophoblasts. During this time, the other two lineages of the blastocyst undergo a dramatic transformation. The PrEnd forms a large primary yolk sac that will later be broken into two, with a smaller ‘secondary yolk sac’ close to the embryonic cells that form the epiblast. Finally, the epiblast forms a disc on top of the PrEnd (Shahbazi and Zernicka-Goetz, 2018; Ross and Boroviak, 2020).

A most important development during this period is the formation of a number of membranes that surround the embryo (Sadler, 2011). From the outside inward, derivatives of the TE interact with extraembryonic mesoderm to form the chorion, which will surround and protect the embryo. The embryonic cells form an epithelial disc, the epiblast, that is tightly apposed to the proximal region of the primitive endoderm and differentiates a dome-like structure, creating the amniotic cavity that will keep the embryo and the fetus in a liquid environment. By the end of the second week, the structures associated with the embryonic cells create an asymmetric environment in the epiblast. At one end, the PrEnd differentiates a thickened epithelium that probably corresponds to the anterior visceral endoderm (AVE) of mice that will determine the anterior pole of the embryonic cells. At the opposite end, a derivative of the extraembryonic mesoderm, the connecting stalk, identifies the posterior pole and will serve as a guide for the development of the allantois and, much later, the umbilical cord. Its origin, as much of the extraembryonic mesoderm before gastrulation, is not clear; It could be PrEnd, embryo, or both. A most significant feature of this period in human embryos is the emergence of the amnion from the epiblast, something that in mice happens at the same time as gastrulation (Luckett, 1975; Chuva de Sousa Lopes *et al.*, 2022; Tam *et al.*, 2026).

By the end of the second week, the organization of the embryo and the extraembryonic membranes is in place, and, approximately on day 14 from fertilization, gastrulation is initiated with the emergence of the primitive streak at the pole marked by the connecting stalk (Rahilly and Müller, 2001; Ghimire *et al.*, 2021; Sheng *et al.*, 2021). Our current knowledge of this process has relied on the Carnegie collection and histological analysis, as well as comparative analysis with embryos of other mammals, particularly monkeys.

During the third and fourth weeks of development, the epiblast increases dramatically in cell numbers, and germ layers emerge simultaneously with a system of coordinates that positions the primordia of the different tissues and organs, laying down the body plan (Rahilly and Müller, 2001). This overview suggests that the early stages of human development, from zygote to body plan, can be divided into three phases, each lasting a week. The first one relates to the formation of the blastocyst, the second one to its implantation into the uterus, and, finally, the third one to gastrulation.

## Working with human embryos

The development of techniques to grow the zygote into a blastocyst *in vitro* (Steptoe *et al.*, 1971) opened up an opportunity to explore the development of human embryos beyond the valuable but coarse descriptions present in medical textbooks, largely derived from studies of pathological samples, and the embryos in the Carnegie and Kyoto collections. In particular, the availability, with consent, of zygotes from surplus ART treatment has allowed an experimental approach to blastocyst formation. This has provided insights into the cellular and molecular events that take place during this period (Rossant and Tam, 2009; Boroviak and Nichols, 2014; Gerri *et al.*, 2020). In all this work, the mouse embryo has remained a reference though. As differences between the two species emerge (Ghimire *et al.*, 2021), the need to study human biology with human embryos becomes paramount; these differences are as much in terms of morphology as in the use of conserved genes and, probably, physiology (Gerri *et al.*, 2020).

The formation of the blastocyst is very similar in all mammals and follows the pattern described above, albeit with different timings for different species. In humans and other primates. blastocyst formation is completed in a week, whereas in mouse, it takes about three and a half days (Nakamura *et al.*, 2021). The mechanisms for this timing difference remain unknown. The slow pace of human development is reflected in a delayed onset of zygotic genome activation, two-cell stage in the mouse, four to eight cells in humans (Jukam *et al.*, 2017). By day 6, the resulting structure, the blastocyst, is now at the bottom of the uterus and ready to implant itself into the endometrium.

Both mouse and human cleaving zygotes share the complement of transcriptional regulators associated with blastocyst formation. Lineage segregation follows a similar pattern, with a segregation of TE from ICM around the 8-cell stage being accompanied by the restriction of CDX2 expression to the outer cells and of OCT4 to the ICM. However, in humans, OCT4 remains longer in the TE, suggesting a plasticity that is not very pronounced in mice (Gerri *et al.*, 2023; Skory, 2024). Following this fate decision, the ICM is subdivided into the PrEnd, characterized by the expression of FOXA2 and GATA4, 6, and the embryonic lineages expressing NANOG, SOX2, and OCT4 (Corujo-Simon *et al.*, 2023). The signalling requirements for these decisions, including YAP (Regin *et al.*, 2023) appears to be conserved, although in humans the role of FGF/ERK signalling in this decision is open to discussion as it appears to play a permissive rather than the instructive role it has in mouse (Kuijk *et al.*, 2012; Roode *et al.*, 2012; Simon *et al.*, 2025). During the late stages of blastocyst formation, the TE becomes specialized into polar-cells contacting the embryonic primordium, characterized by high levels of CDX2- and polar-cells largely around the cavity (Xiang *et al.*, 2020; Corujo-Simon *et al.*, 2024).

After implantation of the blastocyst, it is difficult to obtain material for study. Until recently, most of our knowledge of this period and the ensuing gastrulation relied on histological analyses of specific medical cases and specimens of the Carnegie collection (Noe, 2004; Yamada *et al.*, 2006; de Bakker *et al.*, 2016). These have provided valuable information that has guided further studies but, in the era of molecular and cell biology, it has left many questions unanswered.

One solution to this impasse has been provided by the culture of blastocysts *in vitro*. However, while this has been informative for the pre-implantation period, it faces some challenges for the implantation stages. The most important one is the difficulty to reproduce *in vitro* the environment *in vivo*. Associated with this is the low number of blastocysts that implant properly, which complicates the assessment of the outcome of any experiment. How much of the final morphology is due to a damaged blastocyst or to the experiment? In addition, there are ethical and legal challenges in the form of the day 14 (D14) rule. A consequence of the birth of Louise Brown, this rule explicitly prohibits the culture of human embryos beyond the initiation of gastrulation on the 14th day from conception (Cavaliere, 2017; Wallace, 2017; Franklin, 2019).

Notwithstanding these caveats, various attempts have been made to culture human blastocysts to the initiation of the primitive streak using special matrices in 2D or 3D, with varying degrees of success (Deglincerti *et al.*, 2016; Shahbazi *et al.*, 2016; Zheng *et al.*, 2019; Xiang *et al.*, 2020). In all cases, the TE expands and differentiates into cytotrophoblasts and syncytiotrophoblasts, but these cells tend to overgrow, the chorion does not form properly, and the primitive endoderm does not differentiate correctly. The most successful explants have been in 3D and have taken human blastocysts to the time of gastrulation. Although in all cases culture has been stopped to fulfil the D14 rule, inspection of the resulting structures suggests they would not have progressed much further on their own. Similar experiments with blastocysts from non-human primates (NHP) (Ma *et al.*, 2019; Niu *et al.*, 2019; Gong *et al.*, 2023; Zhai *et al.*, 2023) have fared better and developed, albeit with defects, to the D28.

These experiments have led to an ongoing discussion about whether to extend the D14 rule to day 28 or beyond (Hyun *et al.*, 2016; Appleby and Bredenoord, 2018; Greely, 2022). We are in favour of these changes, but it is important that the morphology of the structures is closely monitored so as not to overclaim. Groups of cells will grow *in vitro* and express programs of gene expression associated with gastrulation, as shown in adherent culture, without this representing embryos. An additional issue that needs to be reckoned with in these studies is the large rate of attrition of blastocyst development. This means that if the culture of a blastocyst does not progress through the second or third week, it will be unclear whether this is due to natural causes—the blastocyst would have failed *in vivo*—or to experimental issues.

**Figure 2.**
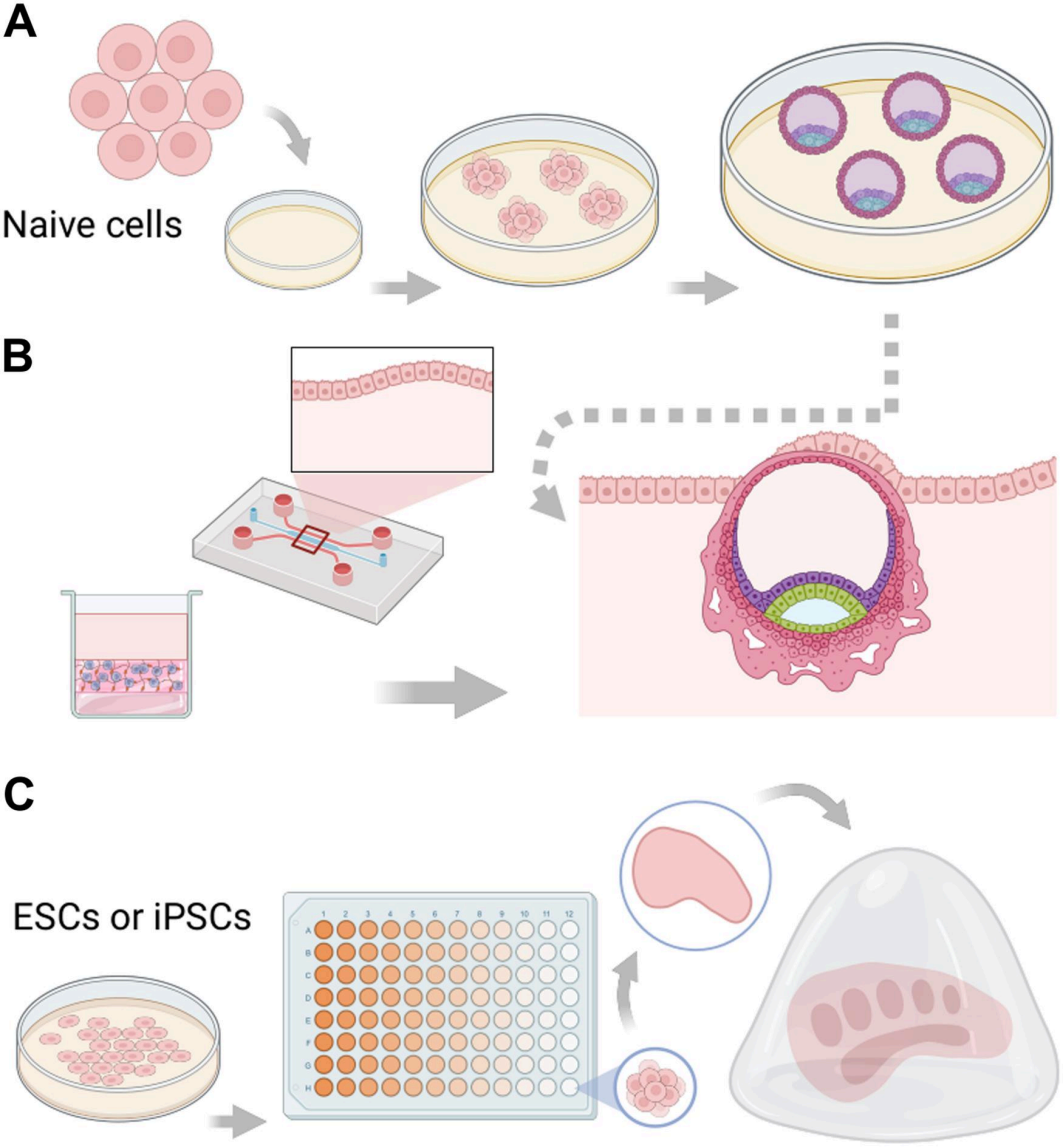
**Examples of current stem cell-based embryo models**. For details, see text. (**A**) Pluripotent and extraembryonic stem cells are aggregated in precise numbers and under defined conditions to generate blastoids, models of blastocyst with the three lineages: embryo (bluish/green), trophectoderm (crimson), and the primitive endoderm (purple). (**B**) Bioengineered endometrial organoids can be used as a substrate to mimic the implantation stages by combining them with blastoids or, in some instances, with blastocysts. The embryonic lineage, differentiates the epiblast with the amnion (green), while the trophectoderm (light crimson) and the primitive endoderm (purple) differentiate and expand (**C**) embryonic (ESCs) or induced pluripotent stem cells (iPSCs) are used to generate gastruloids, Stem cell-based embryo models of gastrulation and body plan formation that differentiate an axial organization and some tissues, like somites, spinal cord and gut. Created in BioRender. Dias, A. (2026). https://BioRender.com/j379t5q.

The need to study peri- and post-implantation studies is urgent. Over the last few years, the discovery of the self-organizing abilities of PSCs has suggested ways to make progress in this direction without the use of embryos. Two kinds of approaches are being pursued: replicas–mimics of the complete structure—and models—simplified constructs that explore the minimal contribution to a structure. These stem cell-based embryo models (SCBEMs) (Fig. 2) are providing insights into the early stages of development and their applications (Fu *et al.*, 2021; Rosner *et al.*, 2025; Wu and Wang, 2026).

## Pre-implantation replicas: advantages, uses, and limitations

The discovery of PSCs—naïve and primed ESCs and iPSCs—and the development of protocols for their differentiation into specific cell types, opened up opportunities to gain deep insights into mammalian and specifically human development (Keller, 2005; Evans, 2011; Damjanov and Andrews, 2016). At the same time, stem cells were derived for the TE (trophoblast stem cells, TSCs) and the PrE (extraembryonic endoderm stem cells, XEN cells) and created the opportunity to reconstruct blastocysts and embryos *in vitro* (Shahbazi *et al.*, 2019; Weatherbee *et al.*, 2021; Zernicka-Goetz, 2023).

Work with mouse PSCs revealed that combinations of naïve PSCs and TSCs give rise to blastocyst-like structures that, in a small number of cases, also contain PrE (Rivron *et al.*, 2018b). The structures were called ‘blastoids’, and although they are able to initiate implantation, the process does not progress very far. This work opened the possibility of creating blastoids from primate PSCs.

In the case of humans, there are two kinds of PSCs: primed, similar to the mouse equivalent but with potential to generate PGCs, and naïve, which resemble the blastocyst and are similar to mouse naïve cells (Nichols and Smith, 2009). Initial attempts to engineer human blastoids made use of primed PSCs and iPSCs (Liu *et al.*, 2021; Yu *et al.*, 2021). The results were blastoids with structural organization and a composition similar to blastocysts but with spurious cells of more advanced stages. The realization that naïve human PSCs are totipotent, led to the generation of more faithful blastoids from these cells with good representation of TE and better PrE than mouse blastoids (Yanagida *et al.*, 2021; Kagawa *et al.*, 2022). This has now become the standard way to build human blastoids. The benchmarking of these structures has been helped by the development of a global atlas of transcription during blastocyst and blastoid development (Zhao *et al.*, 2025).

Blastoids have allowed a deep understanding of the biology of the blastocyst and, in particular, have led to the study of the mechanisms of implantation. As it is not possible to perform *in vivo* implantations in humans, these experiments have to be performed either on endometrial organoids or synthetic/engineered endometrial tissues. However, it is possible to implant blastoids in NHP. In this case, though the early stages of implantation proceed normally, the embryos do not develop far. In the case of Cynomolgus blastoids, implantation proceeds to gastrulation when the embryo disappears (Li *et al.*, 2023). To circumvent this functional gap, significant advances are being made in the development of models that mimic the endometrium as substrates for the early stages of implantation (Bondarenko and Turco, 2025; Liu *et al.*, 2025). Recent studies reported two independent endometrial models that exhibit many of the features of the endometrium and allow the implantation of blastocysts and blastoids (Mole *et al.*, 2026; Song *et al.*, 2026).

These results suggest that while resembling blastocysts, blastoids are still lacking some molecular or cellular features that would make them compatible with maternal tissues. Some of the reasons might lie in their being derived from PSCs rather than from a zygote. It might be that there are developmental ‘licensing’ events that accrue during cleavage that might be important for later development. In this regard, while blastoids are tools for the study of the blastocyst and the early stages of implantation, they are no substitute for early embryos in the study of the cleavage stages. The recent development of mouse totipotent stem cells that have been shown to be a source of more robust embryoids (Li *et al.*, 2025; Yilmaz *et al.*, 2025), might point a way for future research.

On a practical side, while blastoids continue to be developed, their current version can help address some of the pressing issues in ART. Always in the context of their ability to recapitulate the early stages of implantation, they can be used to optimize media and culture conditions for blastocyst implantation. Along the same lines, they can be used as substrates in toxicology and contraceptive tests. In this regard, progress in the engineering of endometrial organoids is very important. The possibilities were shown recently in the development of a 3D chip that could be used with samples from recurrent implantation failure patients to screen for drugs that improved the implantation rate with both blastocysts and blastoids (Li *et al.*, 2025).

## Peri-implantation SCBEMs

Once the blastocyst implants, it undergoes a series of morphological changes that result in a complex of extraembryonic membranes that will protect the embryo and link it to the mother during pregnancy. For the embryonic cells, the process ends with the onset of gastrulation. While useful, the mouse is not an accurate model for these stages in humans. The mouse blastocyst engages in a superficial attachment to the uterus with little endometrial invasion that contrasts with the deeper insertion that is a hallmark of primates (Yoshinaga, 2018; Dimova *et al.*, 2025).

The reconstruction of these stages from mouse PSCs has centered on putting together TSCs, XEN, and PSCs under different conditions from the ones that result in blastoids (Weatherbee *et al.*, 2023; Zernicka-Goetz, 2023). Specifically, they are combined in different proportions and chemical environments with the significant addition of Matrigel to yield a small number of partial models of the post-implantation epiblast and early gastrulation. In some cases, PSCs are induced to the TE and PrEnd lineages with forced gene expression and used in various combinations. These studies have confirmed the requirement for interactions between TE, PrEnd, and embryonic primordia for the organization of gastrulation (Girgin *et al.*, 2021). In some of these experiments, the models undergo gastrulation and, albeit with some defects—principally in the heart and the neural tube—develop as far as the E8.5 stage, at a low frequency and, though with a yolk sac, without TE derivatives (Amadei *et al.*, 2022; Tarazi *et al.*, 2022).

Similar experiments have been attempted with human PSCs. In this case, perhaps due to the tissue complexity of the human conceptus that emerges during implantation, the combinations of cells result in a number of morphologically diverse structures, none of which reflect the complete structure *in vivo*. The assemblies seem to be very sensitive to experimental conditions, and this explains the diversity. Sometimes the initial stages are recapitulated, in particular the formation of the amniotic cavity, but then the assemblies drift into structures displaying varying associations of PrEnd and Epiblast (Weatherbee *et al.*, 2021; Ai *et al.*, 2023; Pedroza *et al.*, 2023; Hislop *et al.*, 2024; Okubo *et al.*, 2024; Huang *et al.*, 2025). Only rarely do these combinations produce structures resembling the D14 embryo. In two instances, the use of naïve PSCs has produced structures that resemble the D14 embryo with some differentiated TE, a yolk sac, a polarized PrEnd, and an epiblast in which gastrulation is initiated. However, these structures would not progress further (Oldak *et al.*, 2023; Chen *et al.*, 2025).

Surprisingly, under defined experimental conditions, PSCs on their own, can be engineered to produce an epiblast with amnion, primordial germ cells and the start of the process of gastrulation without any of the extraembryonic tissues (Shao *et al.*, 2017a, Shao *et al.*, 2017b, Zheng *et al.*, 2019). In the context of peri-implantation models, these results confirm a modularity of the early stages of mammalian development suggested by studies with mouse PSCs that are highly elaborated in the case of the human embryo. They also suggest that embryonic PSCs can break symmetry spontaneously but that the maintenance of the resulting structures requires interactions between embryonic and extraembryonic lineages (Girgin *et al.*, 2021). Furthermore, these interactions serve to anchor and orient the interactions with the maternal contribution to the development of the embryo.

There is no doubt that this is the most important period to study from the clinical point of view. The difficulty in trying to replicate the events associated with implantation in humans contrasts with the ease with which this can be achieved with mouse systems. It may be that, in the future, the best way to progress in our understanding of the implantation stages will be to combine blastoids with improved endometrial organoids.

Current models allow studies of the early stages of implantation and, in that way, provide a continuum with the blastoid studies, but it is clear that more work is needed to ensure the reliability and faithfulness of these models for practical use.

## Models of gastrulation; laying down of the body plan

At the start of the third week, the appearance of the primitive streak triggers gastrulation, a crucial process that lays down the body plan and is the source of many neonatal pathologies (Rahilly and Müller, 2001; Ghimire *et al.*, 2021; Sheng *et al.*, 2021). However, the study of this crucial stage is severely restricted by the D14 rule (Wallace, 2017; Franklin, 2019). Over the last few years, the emergence of SCBEMs of this period, combined with studies of mouse embryos, has begun to shed light on the molecular and cellular basis of this process (Sheng *et al.*, 2021). Perhaps it is in attempts to recapitulate this stage that the difference between replicas and models is most obvious. As we will see, it is surprisingly possible to recapitulate much of this process without starting from a complete blastocyst or the ensemble of epiblast and extraembryonic tissues. Such reduced models reveal much about autonomous models that come together in the whole embryo.

The initial studies of this process involved the plating of PSCs onto micropatterns and the application of signalling molecules associated with the initial stages of gastrulation (Warmflash *et al.*, 2014). This model has been very useful in understanding the way cells respond to combinations of signals and the way these responses lead to cell sorting (Heemskerk and Warmflash, 2016; Camacho-Aguilar and Warmflash, 2020). However, this model lacks the axial spatial organization and dynamics characteristic of gastrulation.

As in the case of blastoids, human SCBEMs focused on gastrulation take their inspiration from work with mouse PSCs. In this system, combinations of PSCs, TSCs, and XEN have led to the development of epiblast-like structures that go through gastrulation (Amadei *et al.*, 2022; Tarazi *et al.*, 2022). The frequency and robustness of this system has been recently increased with the use of totipotent stem cells (Li *et al.*, 2026b, Yilmaz *et al.*, 2025). In all cases, the epiblast models engage in gastrulation at a high frequency, but progression decreases until, in the end, only a small number of structures resemble E8.5 embryos.

Similar approaches with human cells have succeeded in creating structures that mimic the very early stages of gastrulation faithfully, though at a low frequency (Oldak *et al.*, 2023; Chen *et al.*, 2025). A different approach has used blastoids as starting material and produced structures that attempt gastrulation with varying success and yield a variety of structures displaying partial features of developing embryos (Karvas *et al.*, 2023; Liu *et al.*, 2023; Oura *et al.*, 2025). Surprisingly, Cynomolgus blastoid-like structures, when cultured under defined conditions, develop a primitive streak and complete gastrulation, without the fragmentation of the human systems, but do not engage in the early stages of organogenesis (Li *et al.*, 2026a). At the moment, we do not understand the differences between monkey and human systems.

An extreme minimalist approach to gastrulation has made use of aggregates of PSCs or iPSCs on their own. Initial work with mice revealed that titration of the initial number of cells and control over the culture conditions of the aggregate in 3D leads to polarized structures that mimic the outcome of gastrulation with a similar axial patterning and a spatial organization of the primordia of tissues and organs (van den Brink *et al.*, 2014; Beccari *et al.*, 2018). The model has been extended to human primed PSCs (Moris *et al.*, 2020). These structures have been called ‘gastruloids’ and develop in large numbers in a robust and reproducible manner. Surprisingly, they do this in the absence of extraembryonic tissues (Martinez Arias *et al.*, 2022; Turner and Martinez Arias, 2024).

An important feature of gastruloids is that they reveal a clear modularity of the body plan. Thus, they reveal that the primitive streak is composed of two functional modules, one associated with the anterior body plan, from the brain to the forearm, and a second one associated with axial extension and the rest of the body (Dias *et al.*, 2025).

The autonomous development of different components of the body (somites, spinal cord, heart, brain, hematopoietic niche), parallels the observation from the organoid field that organs, or organ-like structures, can develop autonomously and independently of the body. The implications of this observation are important for tissue engineering. Furthermore, it is surprising that it is possible to obtain post-implantation epiblasts without a blastocyst stage, as it is to obtain a body plan without an epiblast. This raises questions of how these modules operate not only as spatial units but also temporally.

From a practical point of view, gastruloids can be used in a variety of ways. Most significantly, in addition to the insights into the biology of the early embryo, they have been shown to be good substrates for toxicology and drug screening (Mantziou *et al.*, 2021; Marikawa, 2022). They also show promise as a platform for the development of a number of tissues, e.g. somites (Yamanaka *et al.*, 2023), trunk-like structures (Makwana *et al.*, 2025), blood (Neupane *et al.*, 2025; Ragusa *et al.*, 2025), PGCs (Neupane *et al.*, 2025), and disease modelling in early embryos.

The obvious differences that exist between SCBEMs and embryos could be construed as due to artifacts of the *in vitro* culture. An alternative view is that they reveal features of normal development that are hidden in the continuity and seamless organization of an embryo. Examples of these are the separation of morphogenesis and spatial organization of gene expression, the modularity of gastrulation, or the ability of some models to develop an axial organization in the absence of extraembryonic tissues (Turner and Martinez Arias, 2024). Having established their correspondence with features and processes in the embryos, rather than a disadvantage, they play a role in the acceptance of, in particular, models as research proxies for embryos (Rivron *et al.*, 2018a, Rivron *et al.*, 2023).

## The future: standards, regulation, and funding

It is early days in the field of SCBEMs, but progress is gathering pace. One significant finding of these studies that we are still coming to terms with is the modularity of the early embryos, namely that what looks as a deeply integrated system *in vivo* can be decomposed into modules *in vitro*. This property of the system is not only structural—embryonic and extraembryonic elements can develop in the absence of each other, and even components of the embryo develop autonomously—but also has a surprising temporal element: blastoids bypass the cleavage of the zygote and gastruloids the blastocyst stage. We are just starting to understand this key property of development and thinking of ways in which we can harness it for practical applications.

Specifically blastoids, and their ability to mimic early implantation, can be used to improve blastocyst culture media and test compounds that favour implantation and thus aid the development of treatments for early miscarriages (Song *et al.*, 2026; Li *et al.*, 2026b; Mole *et al.*, 2026) and aid the understanding of pre-eclampsia and endometriosis. Gastruloids have the potential to generate disease models for neonatal pathologies (Yamanaka *et al.*, 2023; Ragusa *et al.*, 2025) and have the potential to transform teratology (Marikawa *et al.*, 2020; Marikawa, 2022) and drug discovery and in the exploration of safe contraceptives. There is little question that SCBEMs have the potential to carve paths towards solutions. However, on the way there are two issues that need to be borne in mind.

The first one is the need to aim for high standards. This is particularly necessary when there is a clinical aim in sight. There have been some calls to do this (Martinez Arias *et al.*, 2024), but the effort needs to continue and involve regulatory bodies, editors, and most importantly, we, the scientists. At this moment, there is a need for robust, reproducible models that can be associated with natural structures and used accordingly. This will also require the development of interactions between clinics, companies, and research scientists.

A second issue is regulation. Being a new area of research that involves new perspectives on sensitive ethical issues, the work needs to be closely monitored and proceed under strict regulation. Over the last few years, ISSCR, several national bodies, and combined teams of scientists and ethicists have been navigating the issues raised by SCBEMs and laying down recommendations and regulations that are guiding the field. However, it is a field where technical developments rapidly change what needs to be considered. At the moment, a general consensus is that SCBEMs are not embryos and therefore they should not follow the same regulations as embryos. However, they should have their own oversight, and there are rules for this indeed based on their similarities with embryos, features of concern, and perceived ethical issues (Rivron *et al.*, 2023; Cave, 2025; Moris *et al.*, 2026). This will continue to evolve, but it should evolve in parallel with the research,

In the end, the most important requirement for the development of this field should be specific funding. The historical gap between basic and applied research, the lab and the clinic, academia and the private sector should be narrowed. The interaction will favour the development of solutions, will make research more focused, and help achieve research aims.

## Data Availability

No new data were generated or analysed in support of this review.
